# Supplementary figures and images for: Citalopram exhibits immune-dependent anti-tumor effects by modulating C5aR1+ TAMs
Source: eLife. 2026 Feb 9;14:RP103016. doi: 10.7554/eLife.103016 (PMC12885477; doi:10.7554/eLife.103016)

Figure 1C

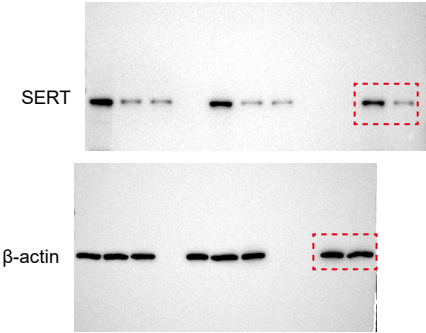

Figure 1D

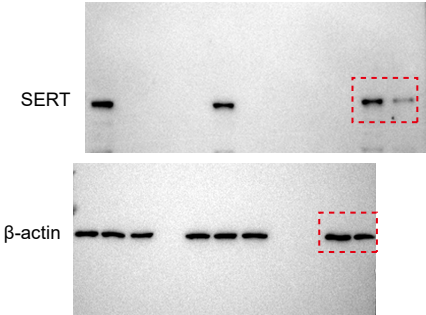

Supplement: Figure 1—source data 1. [file elife-103016-fig1-data1.zip › Figure 1_Source data 1/Figure 1_Source data 1.pdf]

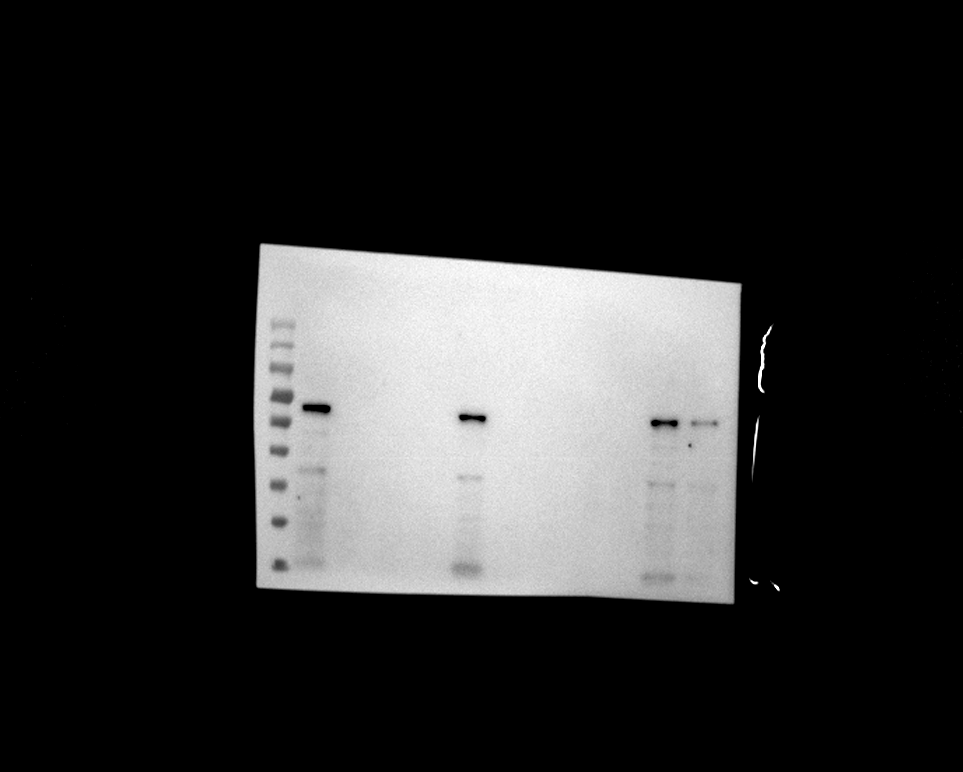

Supplement: Figure 1—source data 2. [file elife-103016-fig1-data2.zip › Figure 1_Source data 2/SERT-Hep53.4.tif]

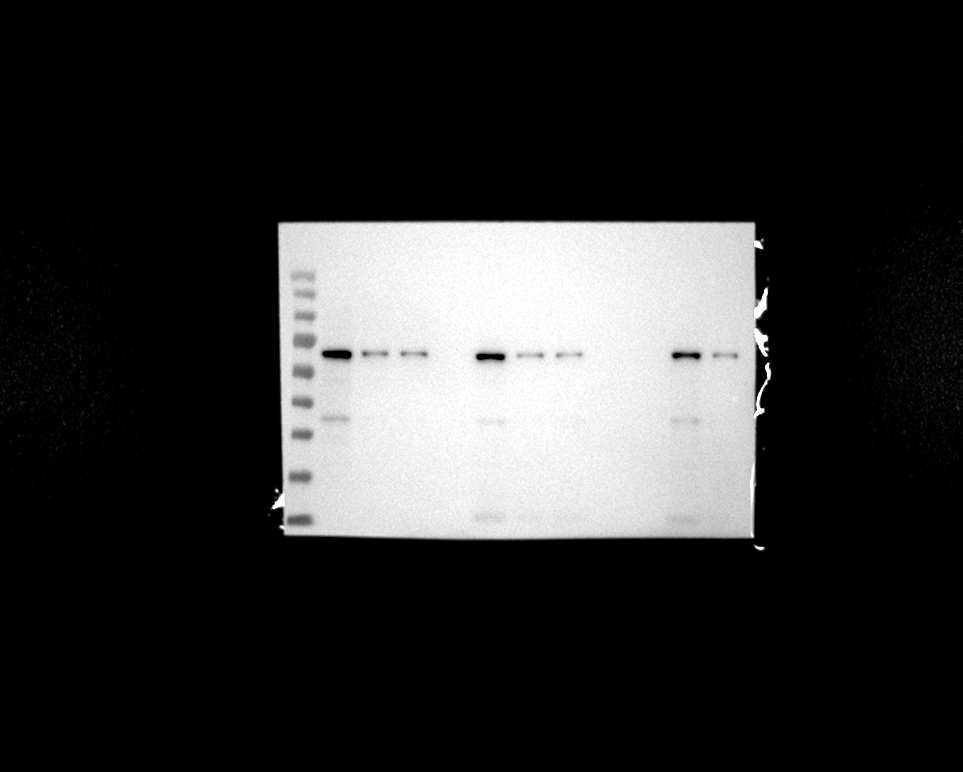

Supplement: Figure 1—source data 2. [file elife-103016-fig1-data2.zip › Figure 1_Source data 2/SERT-Hepa1-6.tif]

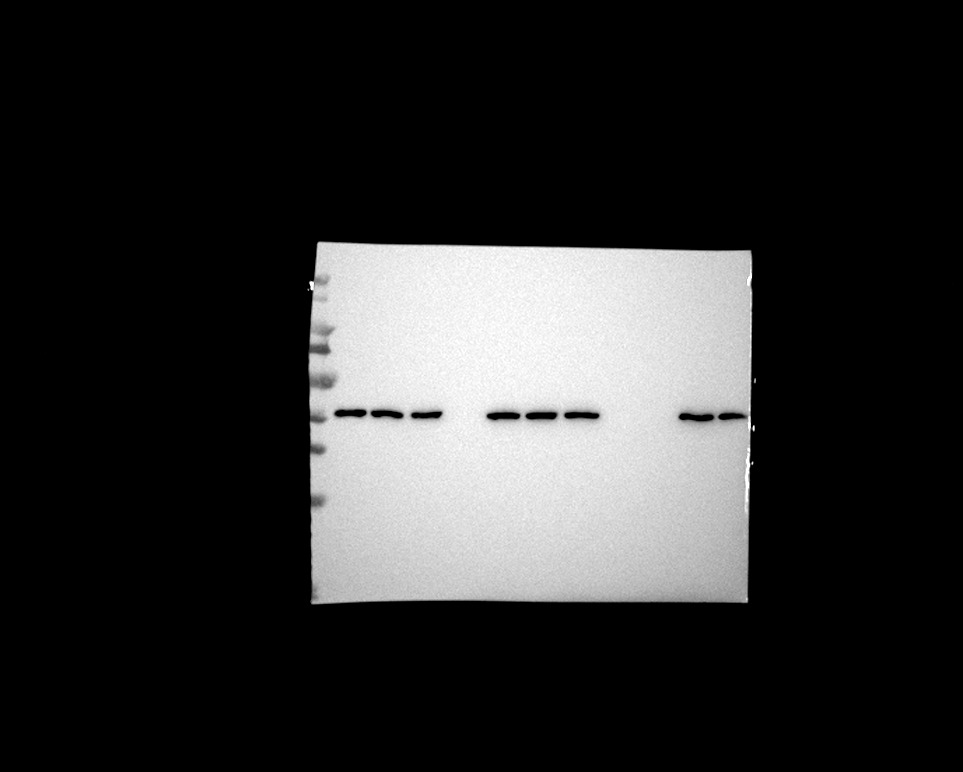

Supplement: Figure 1—source data 2. [file elife-103016-fig1-data2.zip › Figure 1_Source data 2/β-actin-Hep53.4.tif]

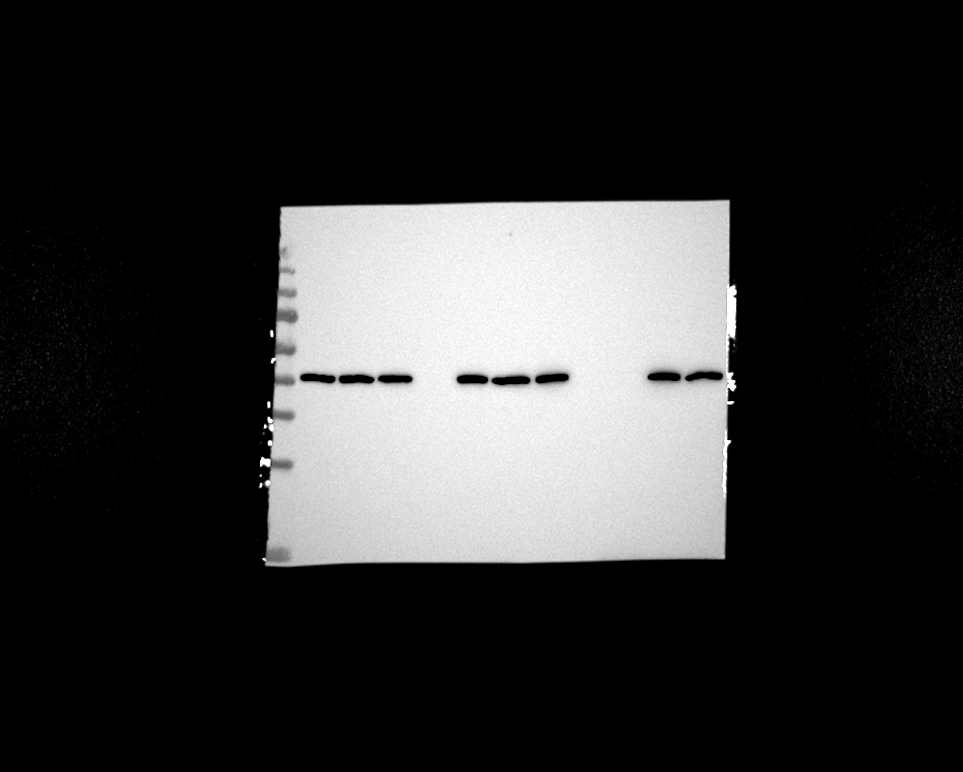

Supplement: Figure 1—source data 2. [file elife-103016-fig1-data2.zip › Figure 1_Source data 2/β-actin-Hepa1-6.tif]

Figure 2F

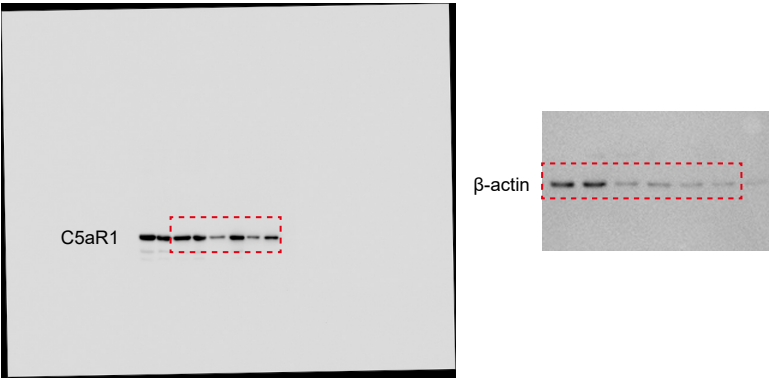

Figure 2G

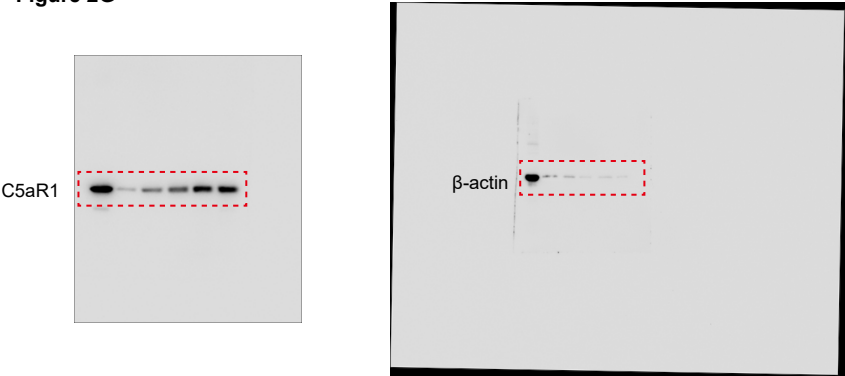

Figure 2J

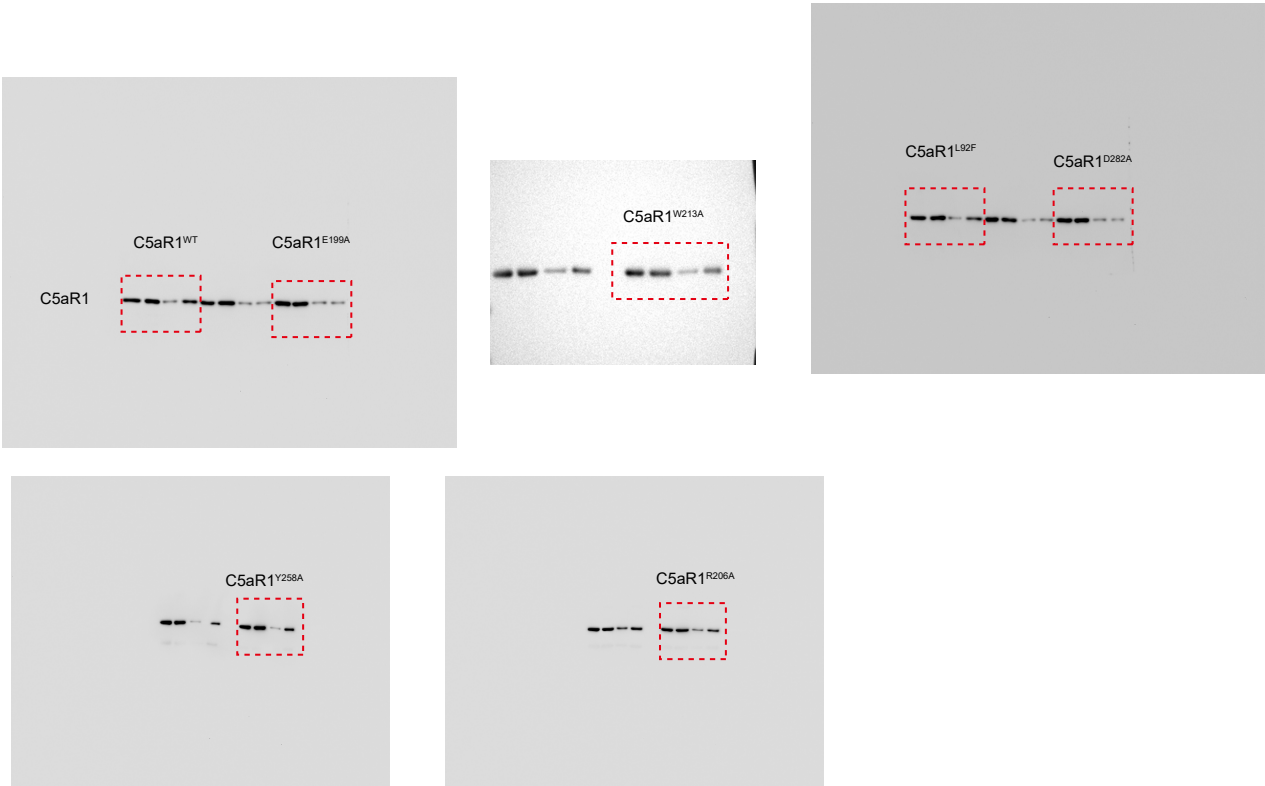

Supplement: Figure 2—source data 1. [file elife-103016-fig2-data1.zip › Figure 2_Source data 1/Figure 2_Source data 1.pdf]

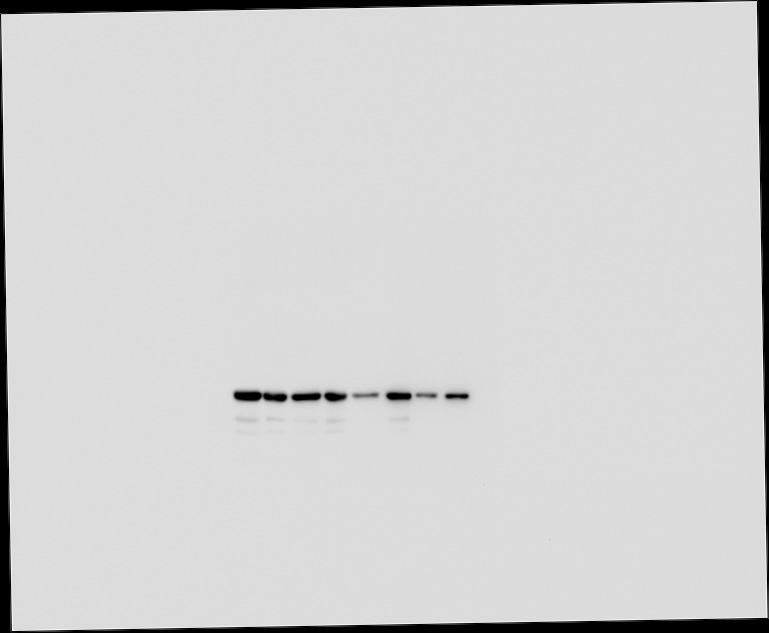

Supplement: Figure 2—source data 2. [file elife-103016-fig2-data2.zip › Figure 2_Source data 2/C5aR1-Figure 2F.jpg]

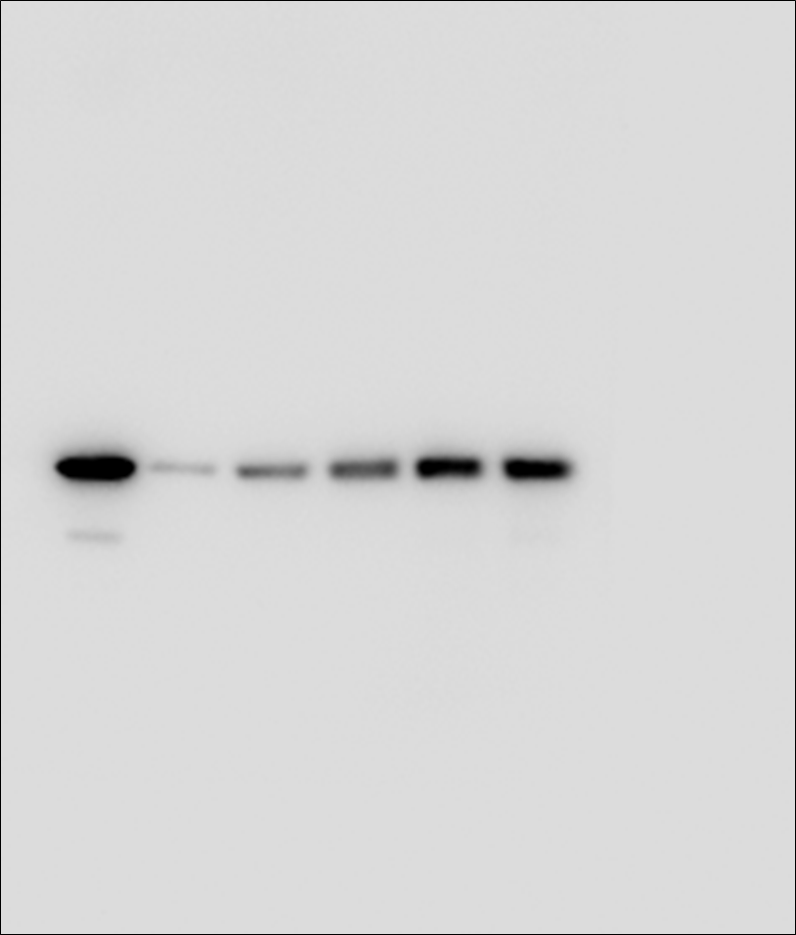

Supplement: Figure 2—source data 2. [file elife-103016-fig2-data2.zip › Figure 2_Source data 2/C5aR1-Figure 2G.tif]

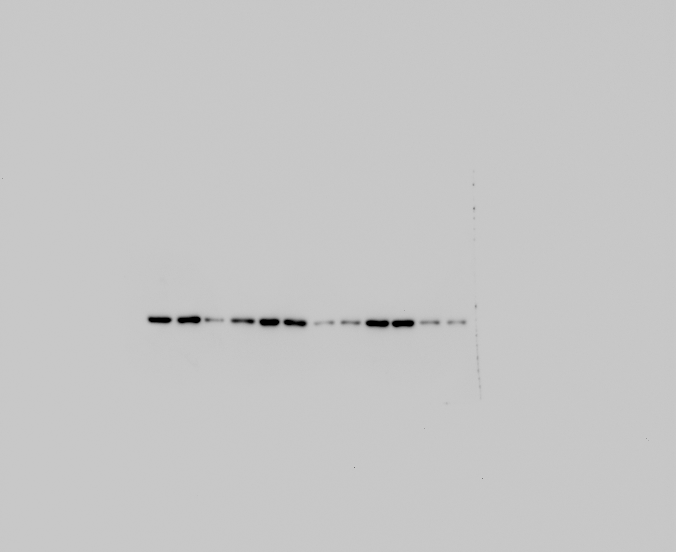

Supplement: Figure 2—source data 2. [file elife-103016-fig2-data2.zip › Figure 2_Source data 2/C5aR1-L92-D282-Figure 2J.tif]

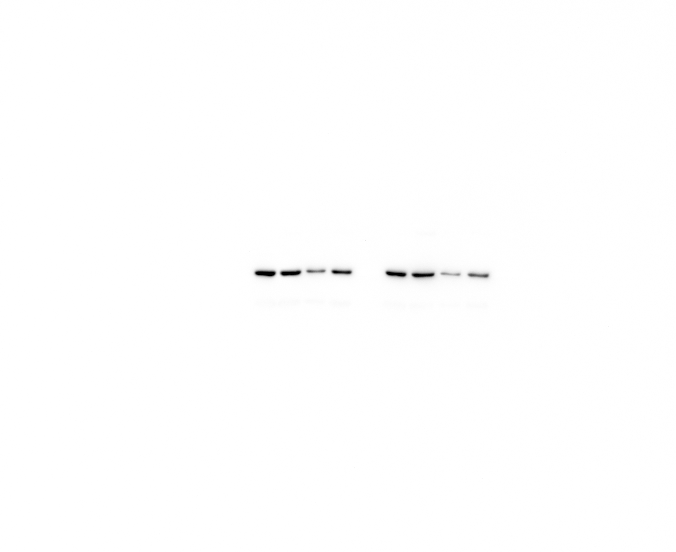

Supplement: Figure 2—source data 2. [file elife-103016-fig2-data2.zip › Figure 2_Source data 2/C5aR1-R206-Figure 2J.tif]

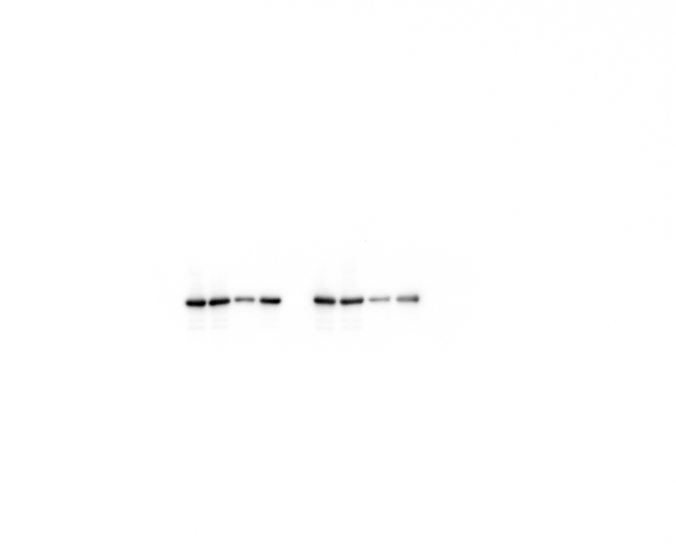

Supplement: Figure 2—source data 2. [file elife-103016-fig2-data2.zip › Figure 2_Source data 2/C5aR1-W213-Figure 2J.tif]

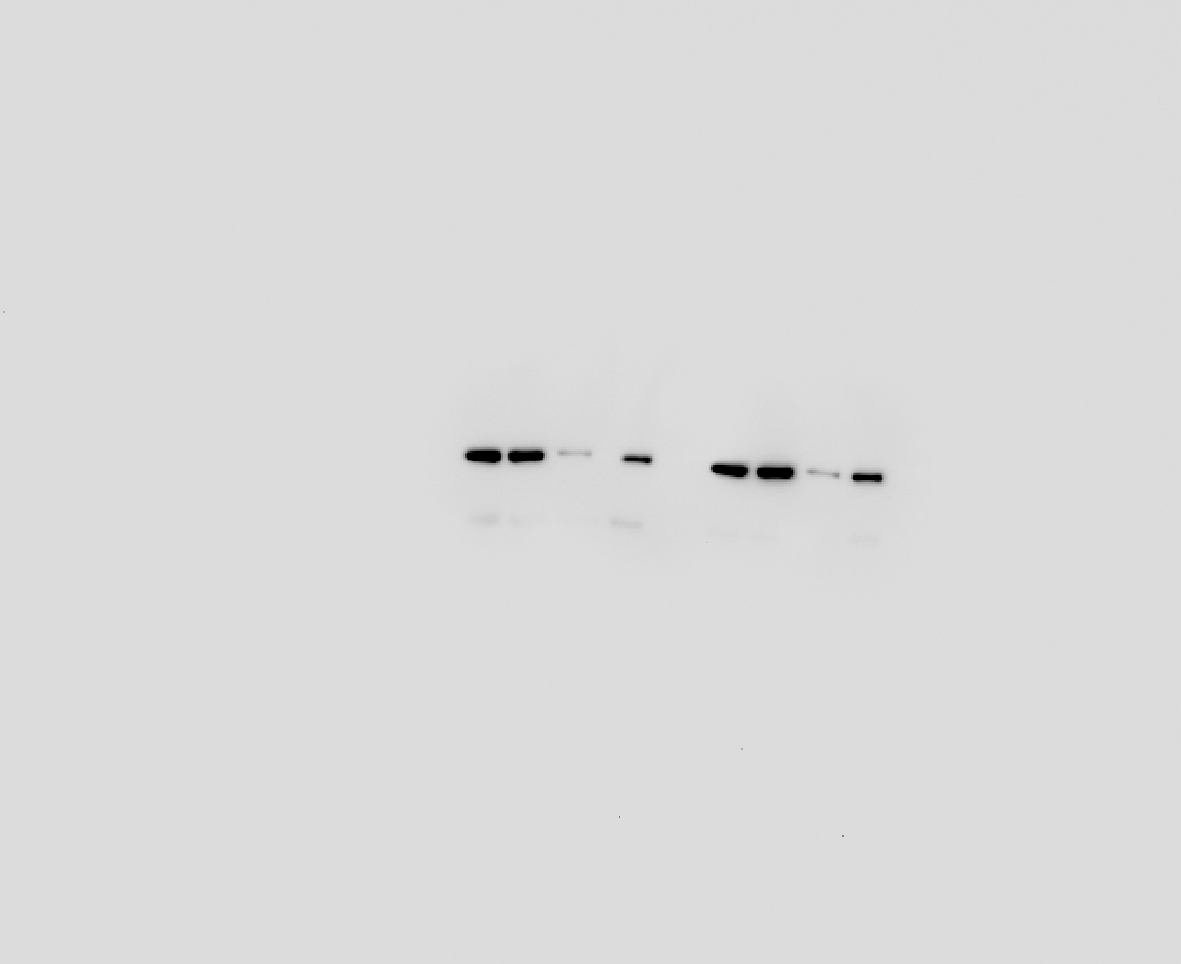

Supplement: Figure 2—source data 2. [file elife-103016-fig2-data2.zip › Figure 2_Source data 2/C5aR1-Y258-Figure 2J.jpg]

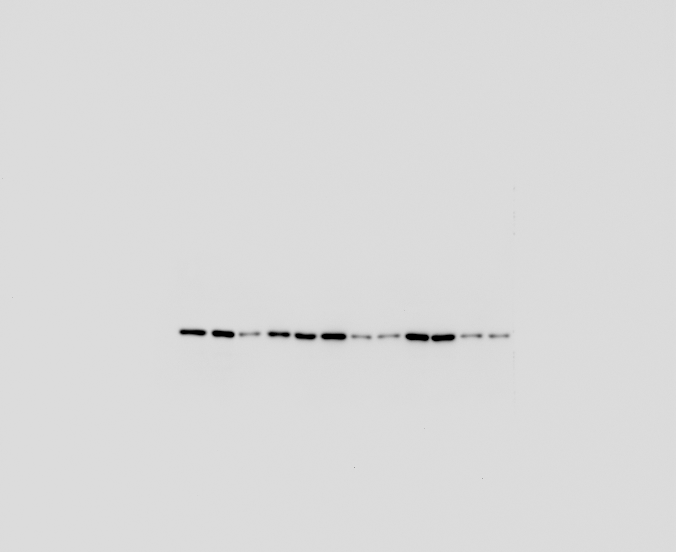

Supplement: Figure 2—source data 2. [file elife-103016-fig2-data2.zip › Figure 2_Source data 2/C5aR1WT-E199-Figure 2J.tif]

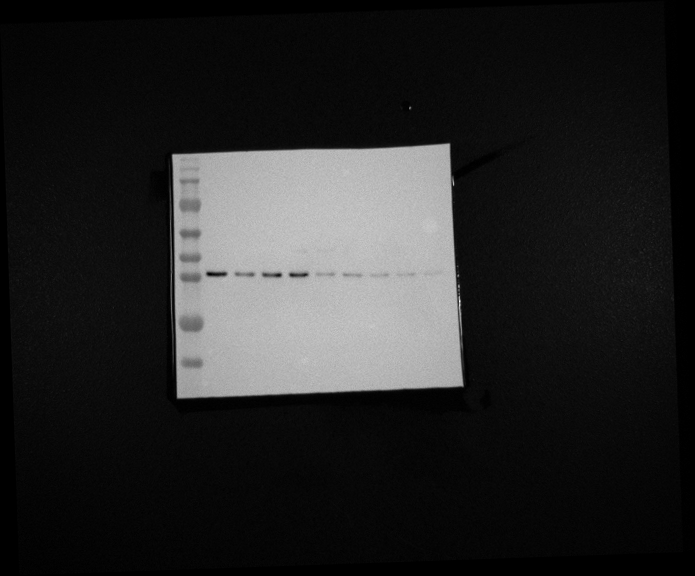

Supplement: Figure 2—source data 2. [file elife-103016-fig2-data2.zip › Figure 2_Source data 2/β-actin-Figure 2F.tif]

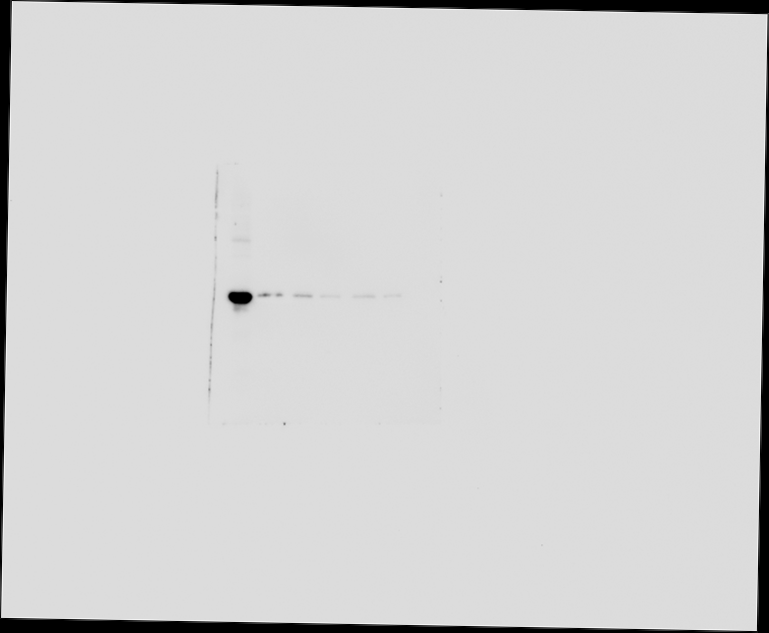

Supplement: Figure 2—source data 2. [file elife-103016-fig2-data2.zip › Figure 2_Source data 2/β-actin-Figure 2G.tif]

Figure 2-figure supplement 2A

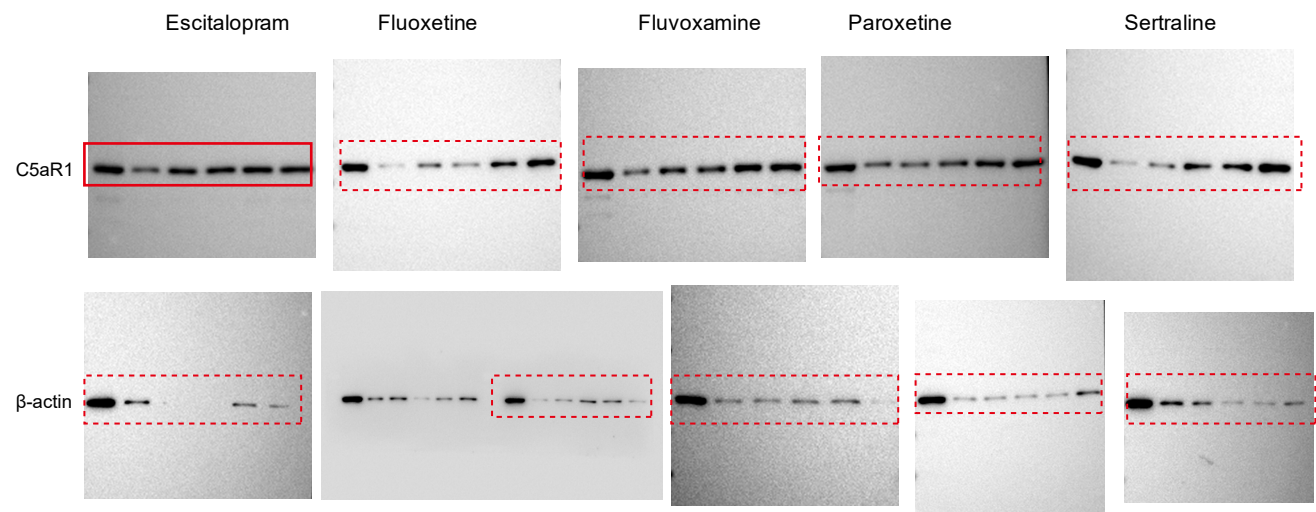

Figure 2-figure supplement 2E

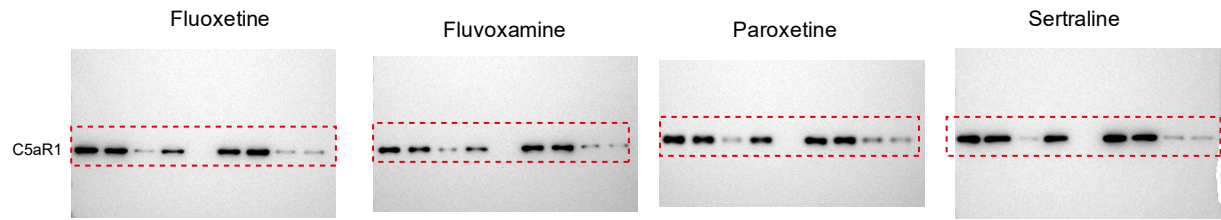

Supplement: Figure 2—figure supplement 2—source data 1. [file elife-103016-fig2-figsupp2-data1.zip › Figure 2-figure supplement 2_Source data 1/Figure 2-figure supplement 2_Source data 1.pdf]

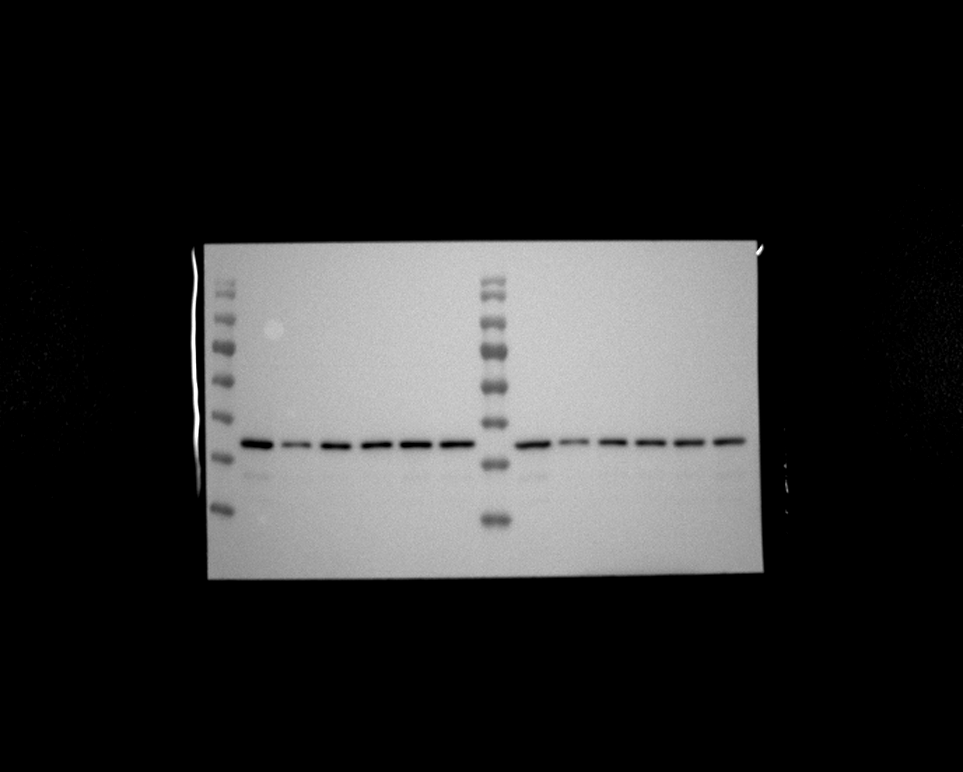

Supplement: Figure 2—figure supplement 2—source data 2. [file elife-103016-fig2-figsupp2-data2.zip › Figure 2-figure supplement 2_Source data 2/Figure 2-figure supplement 2A/C5aR1-Escitalopram.tif]

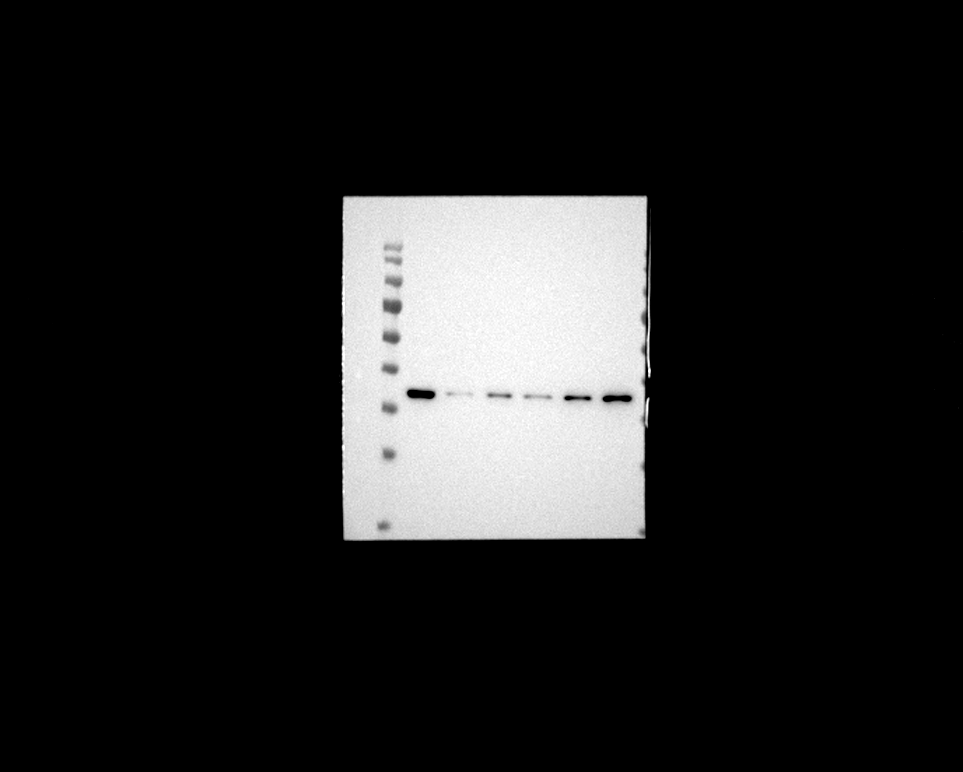

Supplement: Figure 2—figure supplement 2—source data 2. [file elife-103016-fig2-figsupp2-data2.zip › Figure 2-figure supplement 2_Source data 2/Figure 2-figure supplement 2A/C5aR1-Fluoxetine.tif]

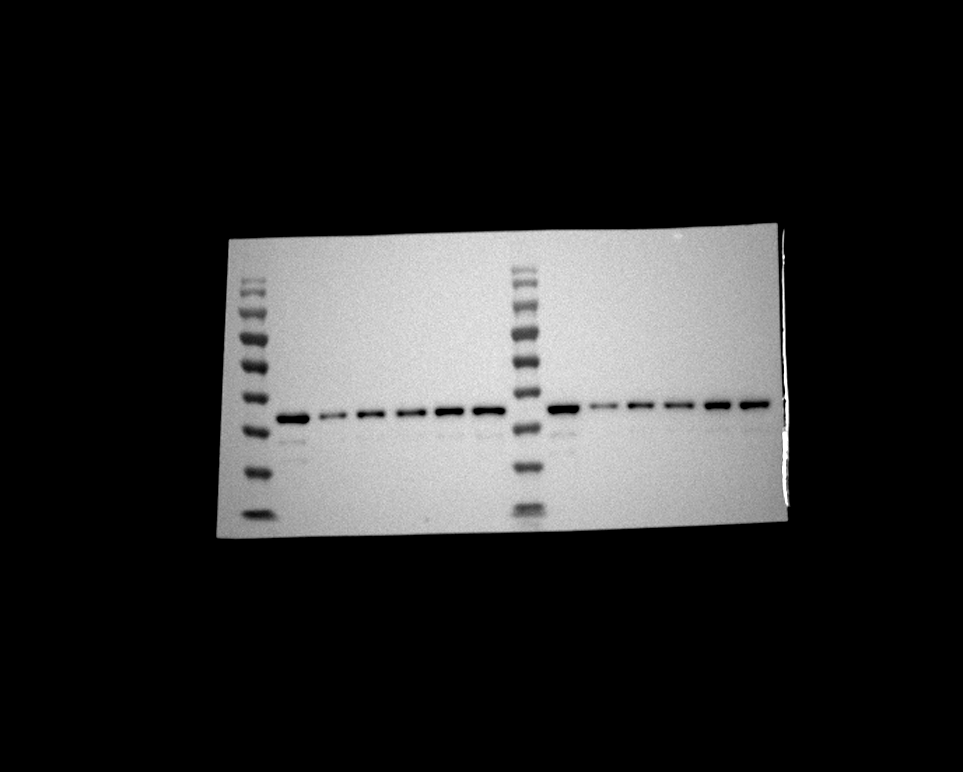

Supplement: Figure 2—figure supplement 2—source data 2. [file elife-103016-fig2-figsupp2-data2.zip › Figure 2-figure supplement 2_Source data 2/Figure 2-figure supplement 2A/C5aR1-Fluvoxamine.tif]

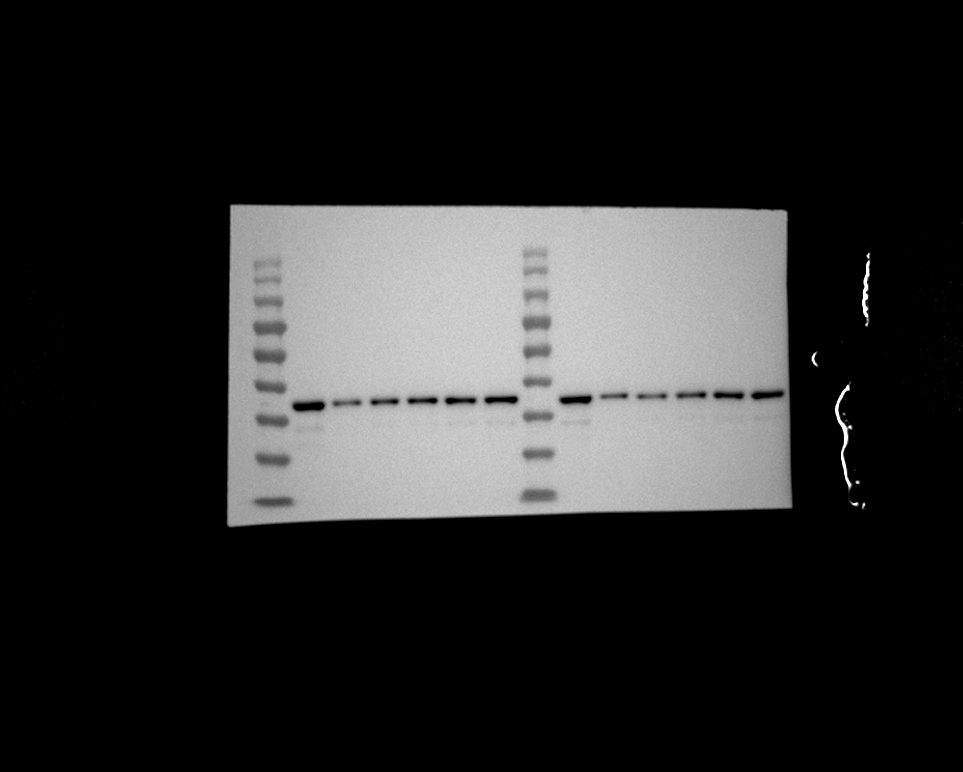

Supplement: Figure 2—figure supplement 2—source data 2. [file elife-103016-fig2-figsupp2-data2.zip › Figure 2-figure supplement 2_Source data 2/Figure 2-figure supplement 2A/C5aR1-Paroxetine.tif]

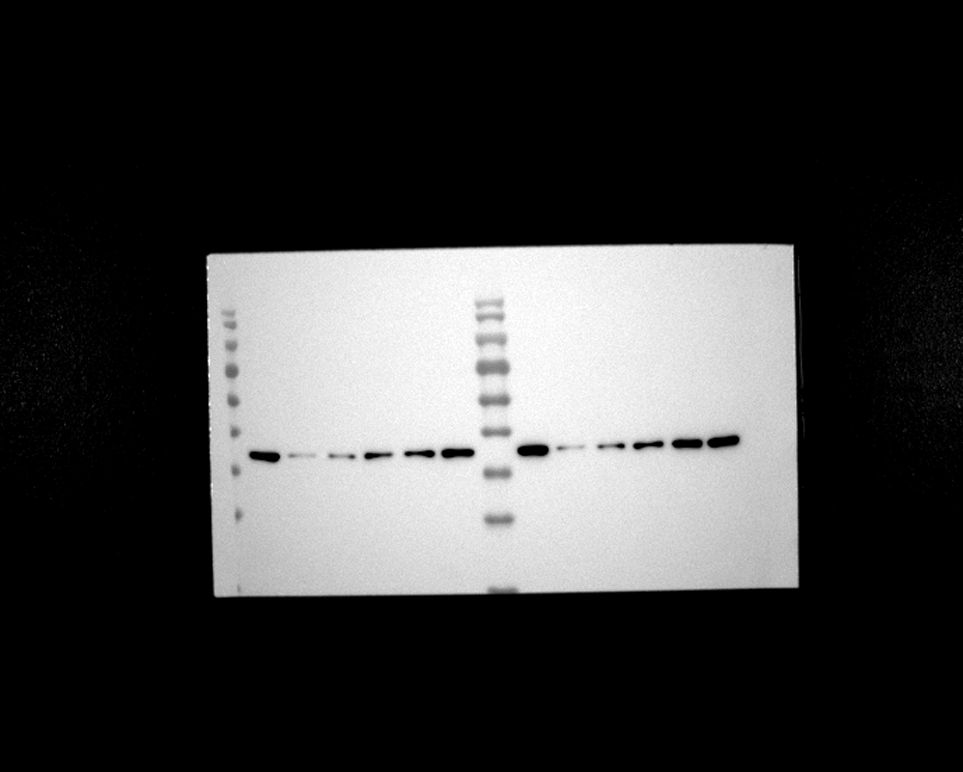

Supplement: Figure 2—figure supplement 2—source data 2. [file elife-103016-fig2-figsupp2-data2.zip › Figure 2-figure supplement 2_Source data 2/Figure 2-figure supplement 2A/C5aR1-Sertraline.tif]

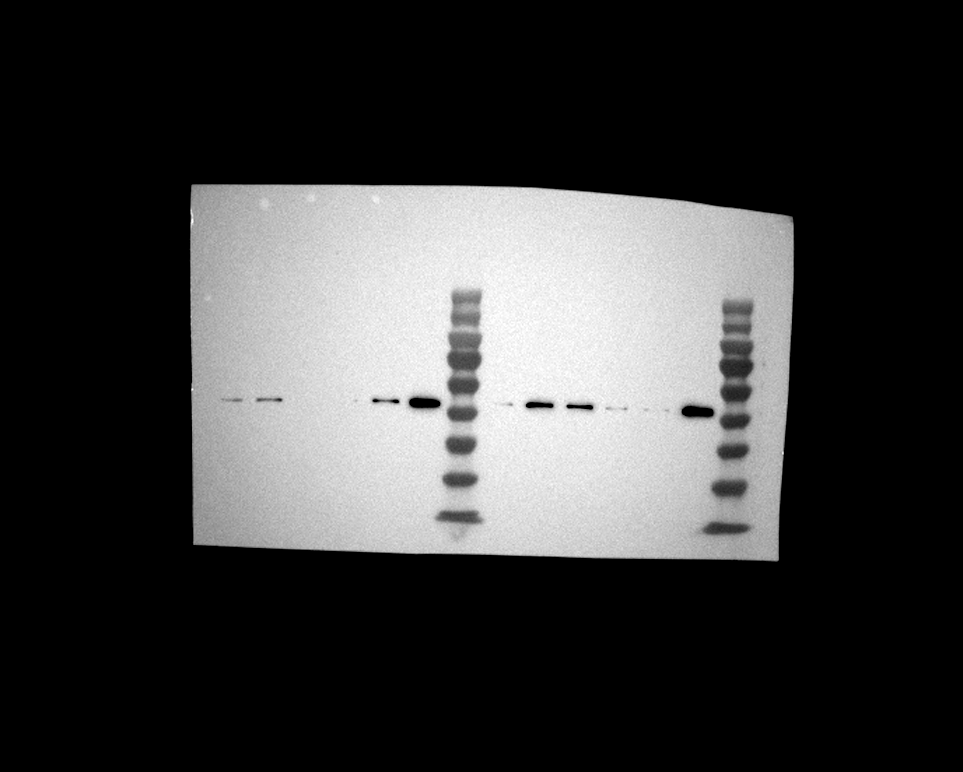

Supplement: Figure 2—figure supplement 2—source data 2. [file elife-103016-fig2-figsupp2-data2.zip › Figure 2-figure supplement 2_Source data 2/Figure 2-figure supplement 2A/β-actin-Escitalopram.tif]

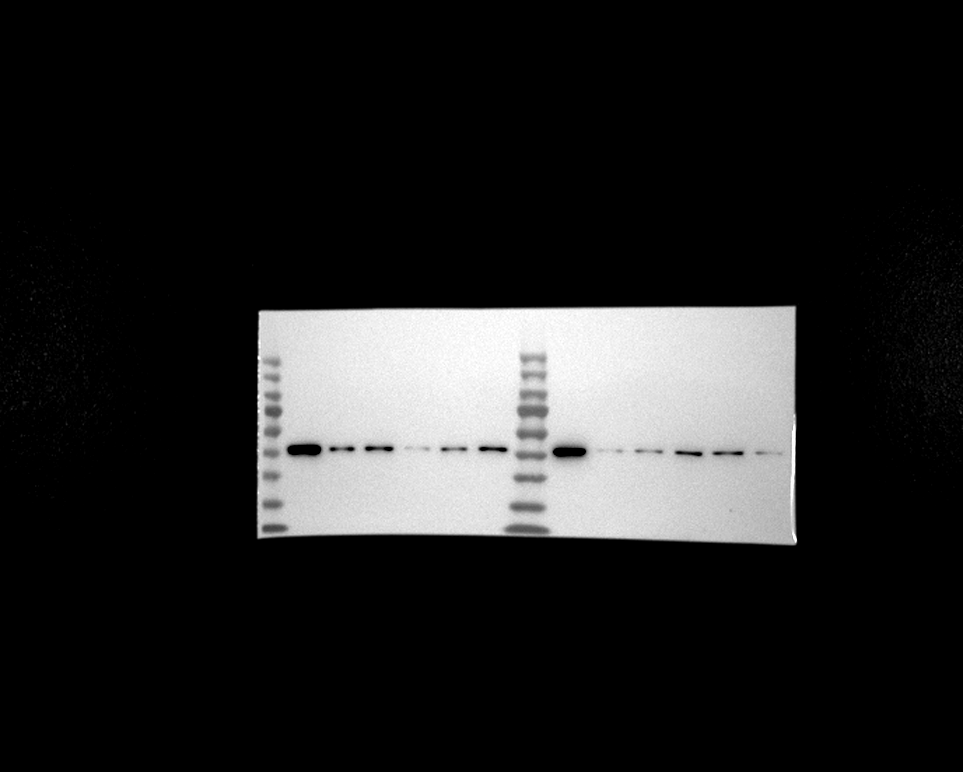

Supplement: Figure 2—figure supplement 2—source data 2. [file elife-103016-fig2-figsupp2-data2.zip › Figure 2-figure supplement 2_Source data 2/Figure 2-figure supplement 2A/β-actin-Fluoxetine.tif]

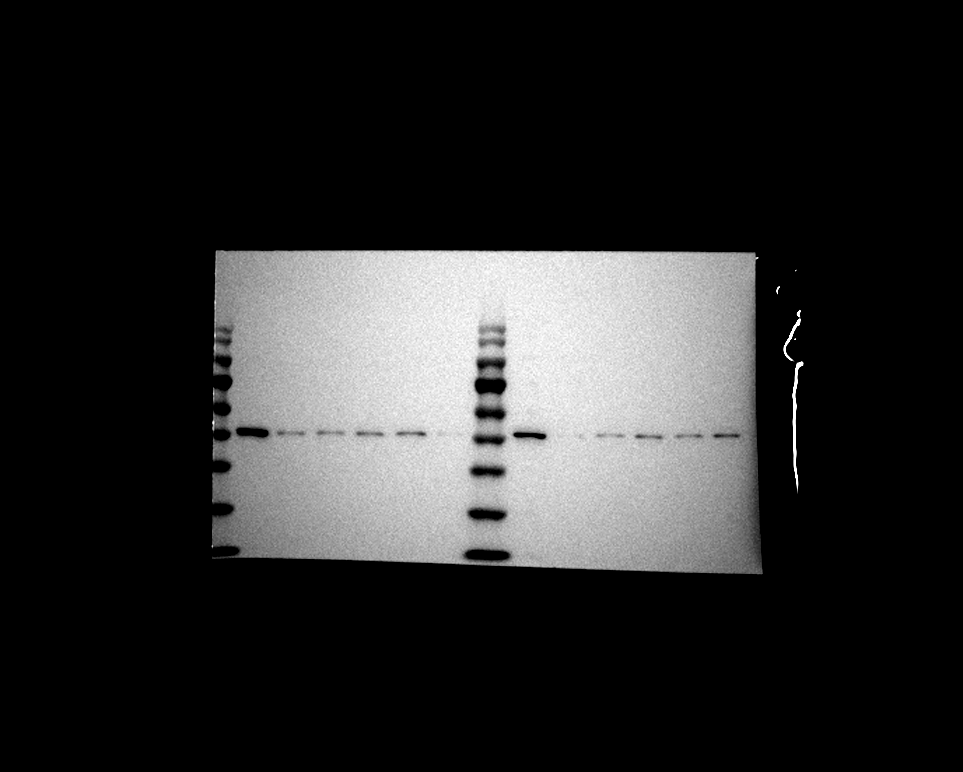

Supplement: Figure 2—figure supplement 2—source data 2. [file elife-103016-fig2-figsupp2-data2.zip › Figure 2-figure supplement 2_Source data 2/Figure 2-figure supplement 2A/β-actin-Fluvoxamine.tif]

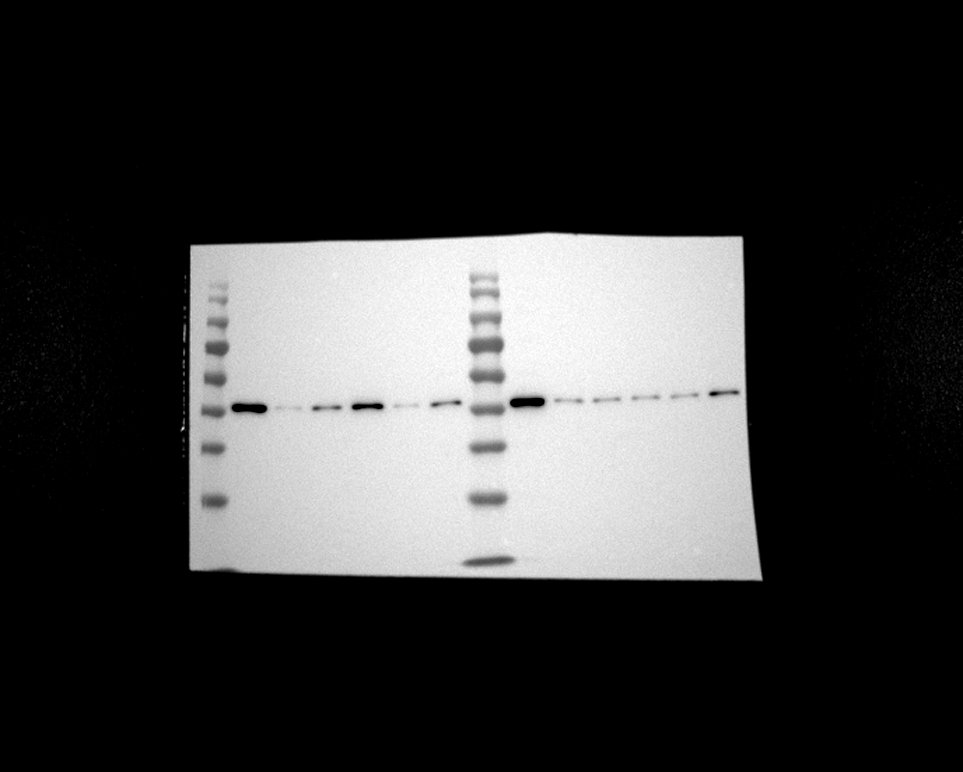

Supplement: Figure 2—figure supplement 2—source data 2. [file elife-103016-fig2-figsupp2-data2.zip › Figure 2-figure supplement 2_Source data 2/Figure 2-figure supplement 2A/β-actin-Paroxetine.tif]

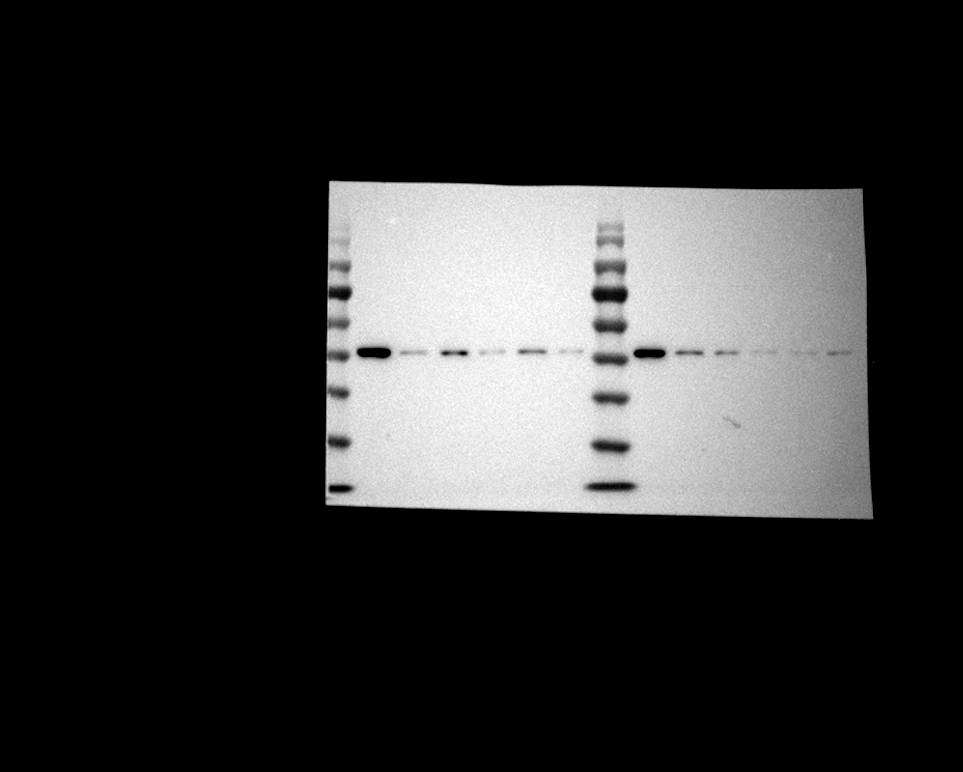

Supplement: Figure 2—figure supplement 2—source data 2. [file elife-103016-fig2-figsupp2-data2.zip › Figure 2-figure supplement 2_Source data 2/Figure 2-figure supplement 2A/β-actin-Sertraline.tif]

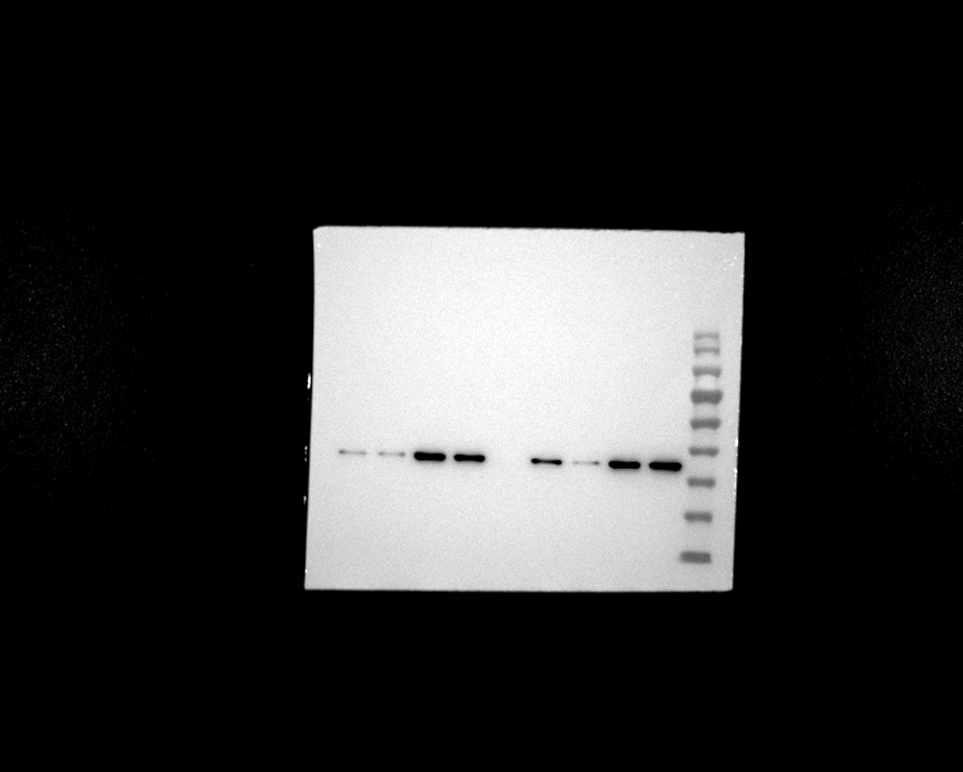

Supplement: Figure 2—figure supplement 2—source data 2. [file elife-103016-fig2-figsupp2-data2.zip › Figure 2-figure supplement 2_Source data 2/Figure 2-figure supplement 2E/C5aR1-Fluoxetine.tif]

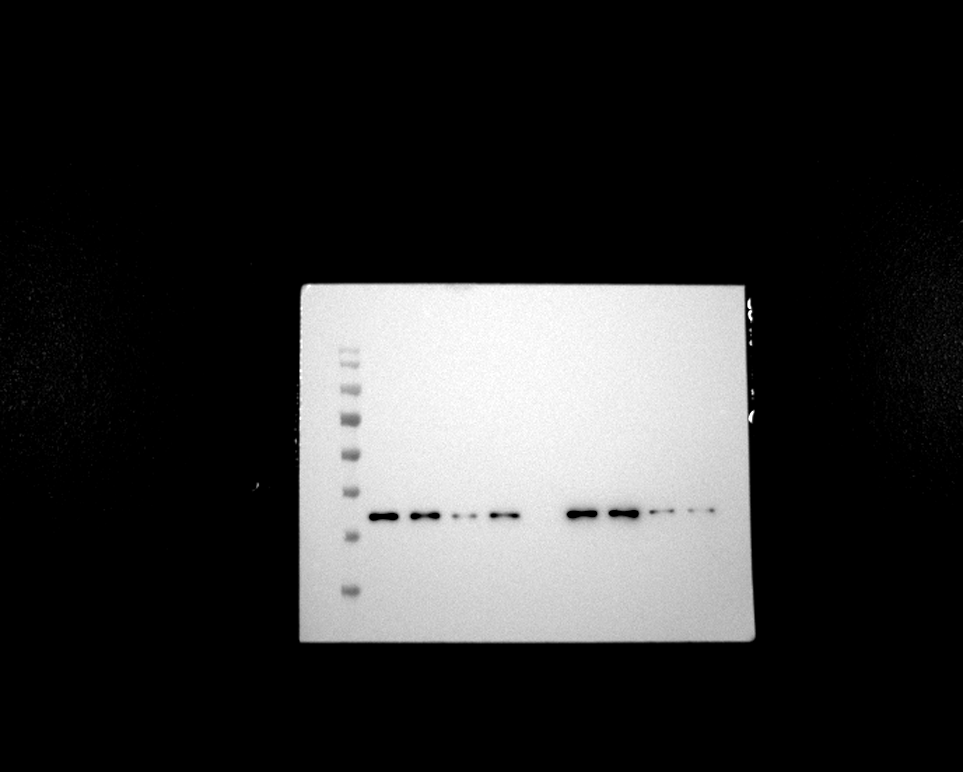

Supplement: Figure 2—figure supplement 2—source data 2. [file elife-103016-fig2-figsupp2-data2.zip › Figure 2-figure supplement 2_Source data 2/Figure 2-figure supplement 2E/C5aR1-Fluvoxamine.tif]

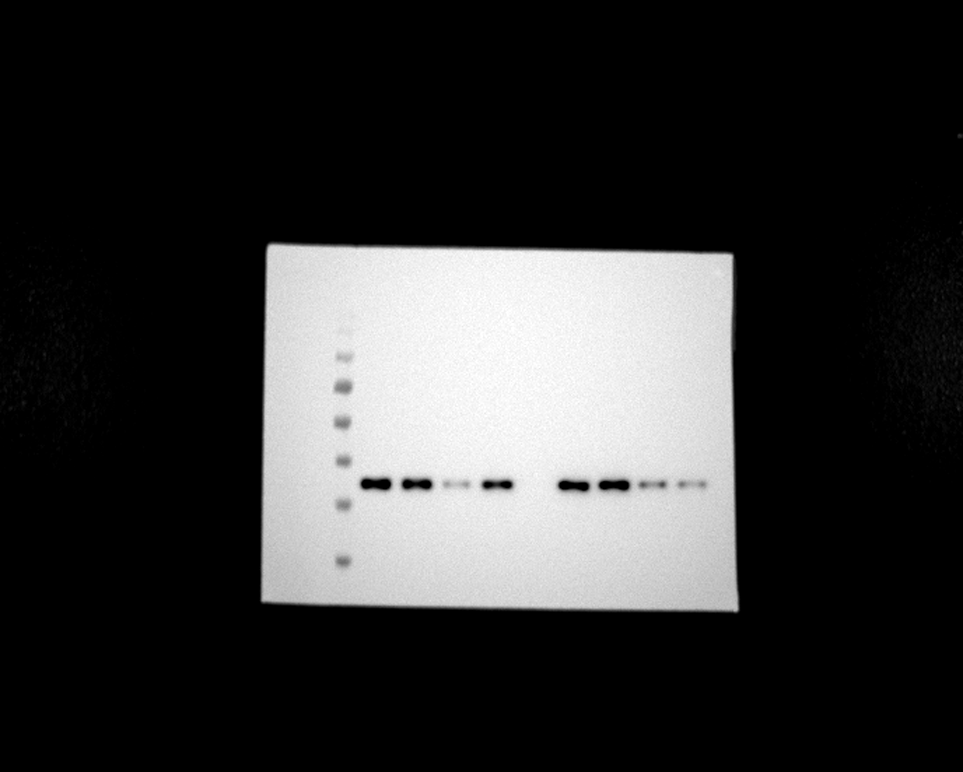

Supplement: Figure 2—figure supplement 2—source data 2. [file elife-103016-fig2-figsupp2-data2.zip › Figure 2-figure supplement 2_Source data 2/Figure 2-figure supplement 2E/C5aR1-Paroxetine.tif]

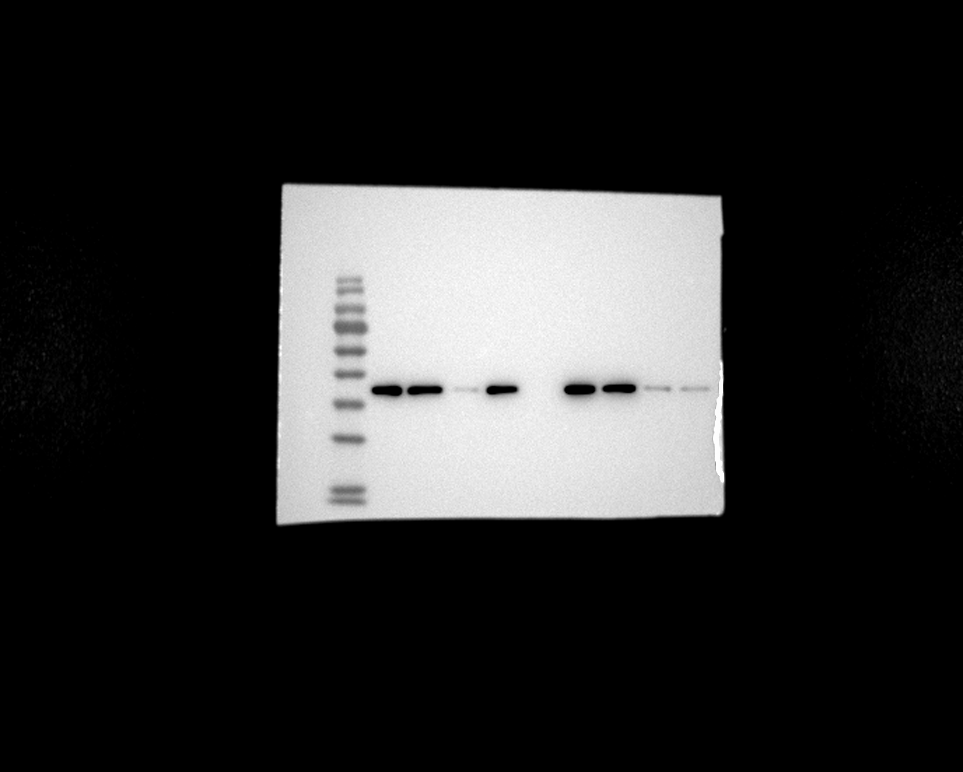

Supplement: Figure 2—figure supplement 2—source data 2. [file elife-103016-fig2-figsupp2-data2.zip › Figure 2-figure supplement 2_Source data 2/Figure 2-figure supplement 2E/C5aR1-Sertraline.tif]

**Figure 3A**

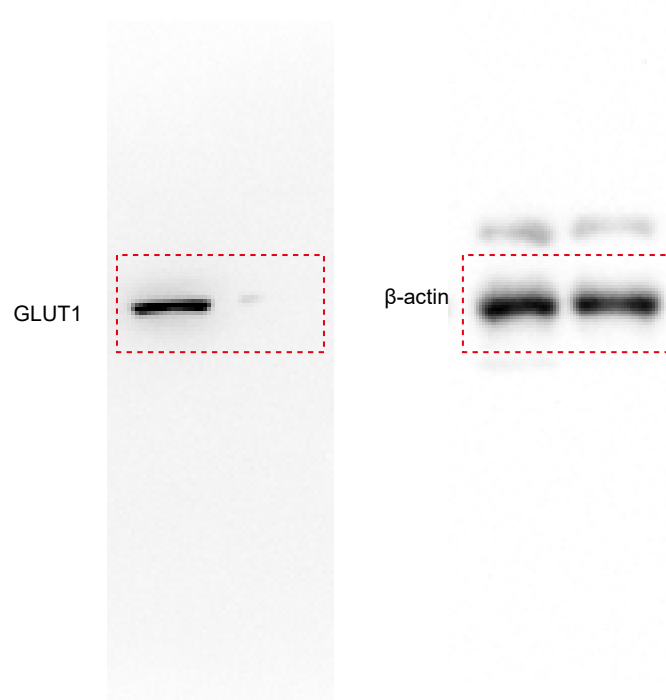

Supplement: Figure 3—source data 1. [file elife-103016-fig3-data1.zip › Figure 3_Source data 1/Figure 3_Source data 1.pdf]

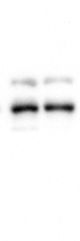

Supplement: Figure 3—source data 2. [file elife-103016-fig3-data2.zip › Figure 3_Source data 2/Hepa1-6-beta-actin.tif]

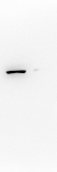

Supplement: Figure 3—source data 2. [file elife-103016-fig3-data2.zip › Figure 3_Source data 2/Hepa1-6-GLUT1.tif]

Figure 3-figure supplement 2

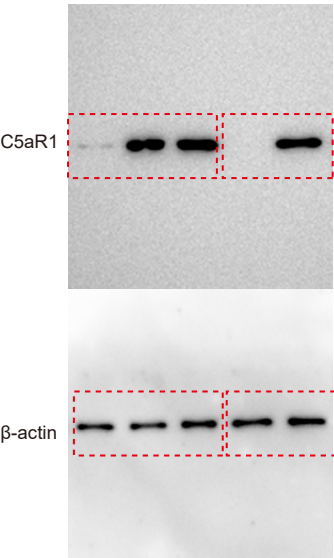

Supplement: Figure 3—figure supplement 2—source data 1. [file elife-103016-fig3-figsupp2-data1.zip › Figure 3-figure supplement 2_Source data 1/Figure 3-figure supplement 2.pdf]

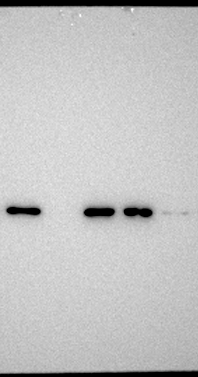

Supplement: Figure 3—figure supplement 2—source data 2. [file elife-103016-fig3-figsupp2-data2.zip › Figure 3-figure supplement 2_Source data 2/C5aR1.tif]

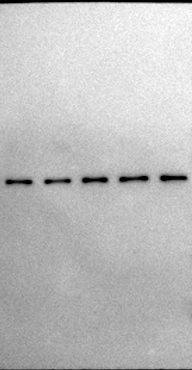

Supplement: Figure 3—figure supplement 2—source data 2. [file elife-103016-fig3-figsupp2-data2.zip › Figure 3-figure supplement 2_Source data 2/β-actin.tif]

Figure 3-figure supplement 4

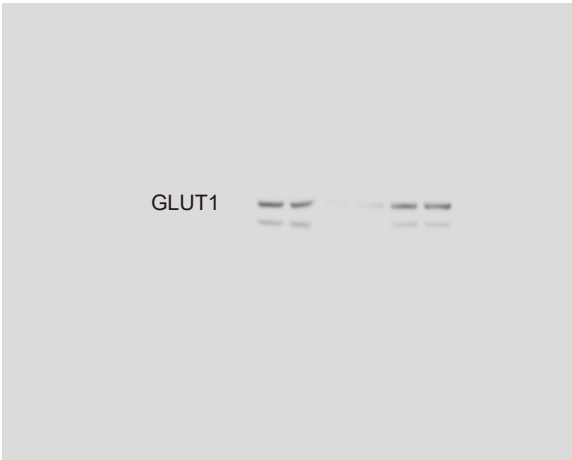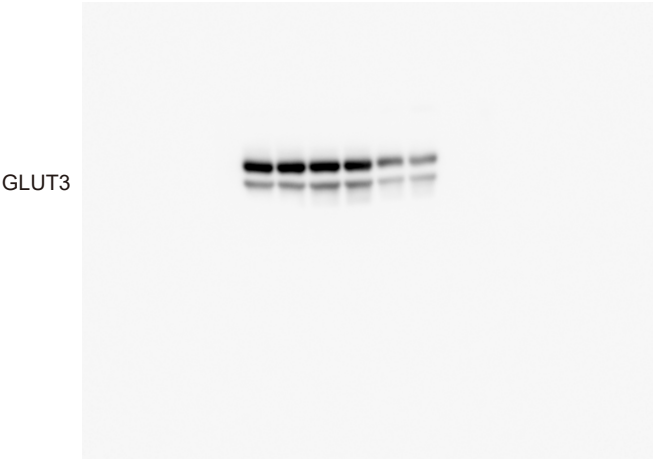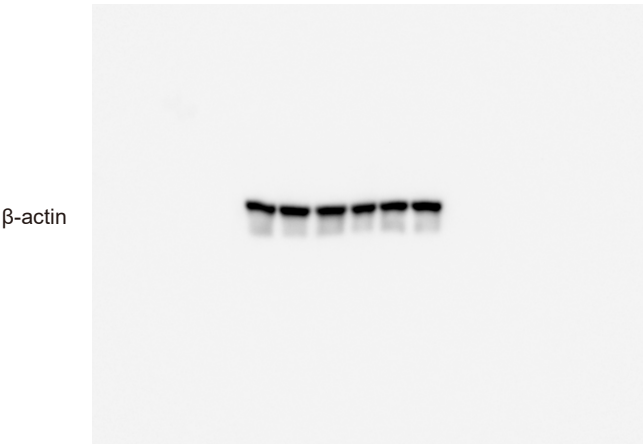

Supplement: Figure 3—figure supplement 4—source data 1. [file elife-103016-fig3-figsupp4-data1.zip › Figure 3-figure supplement 4_Source data 1/Figure 3-figure supplement 4.pdf]

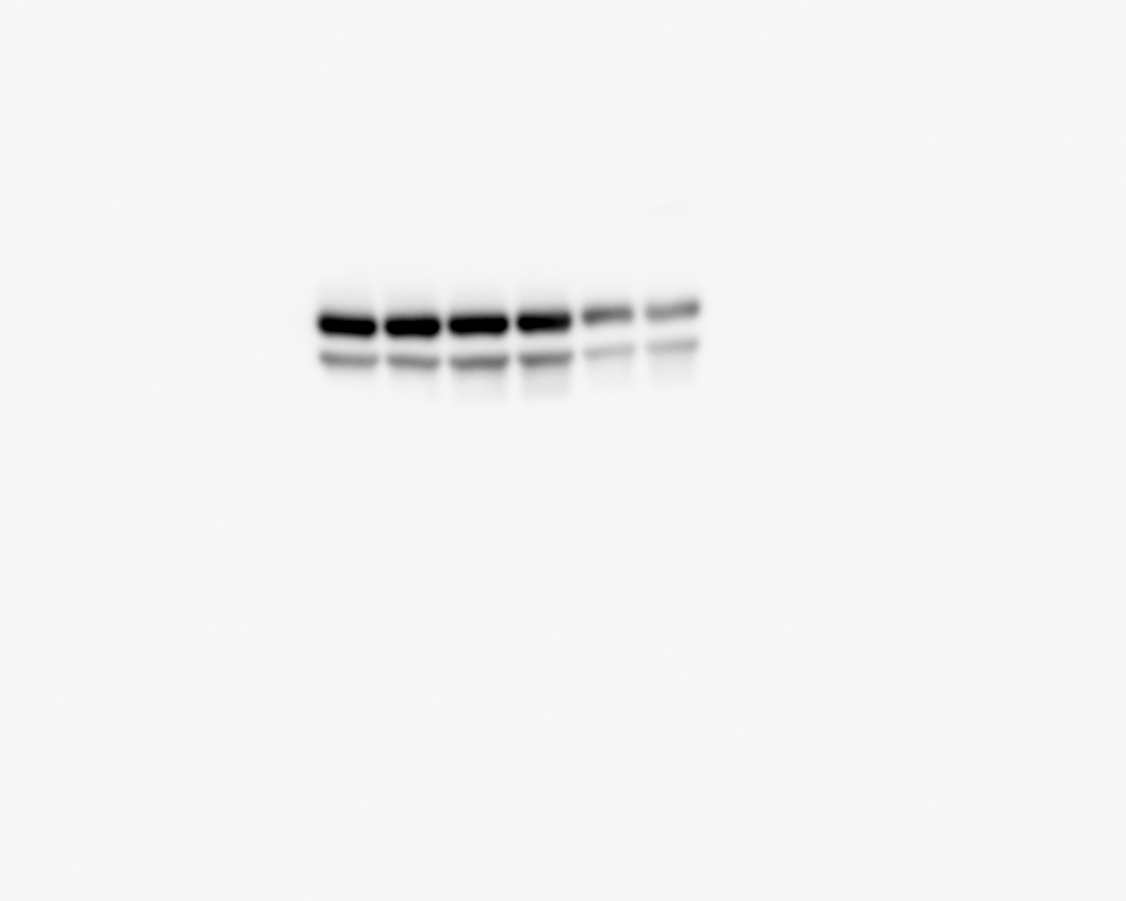

Supplement: Figure 3—figure supplement 4—source data 2. [file elife-103016-fig3-figsupp4-data2.zip › Figure 3-figure supplement 4_Source data 2/GLUT3.tif]

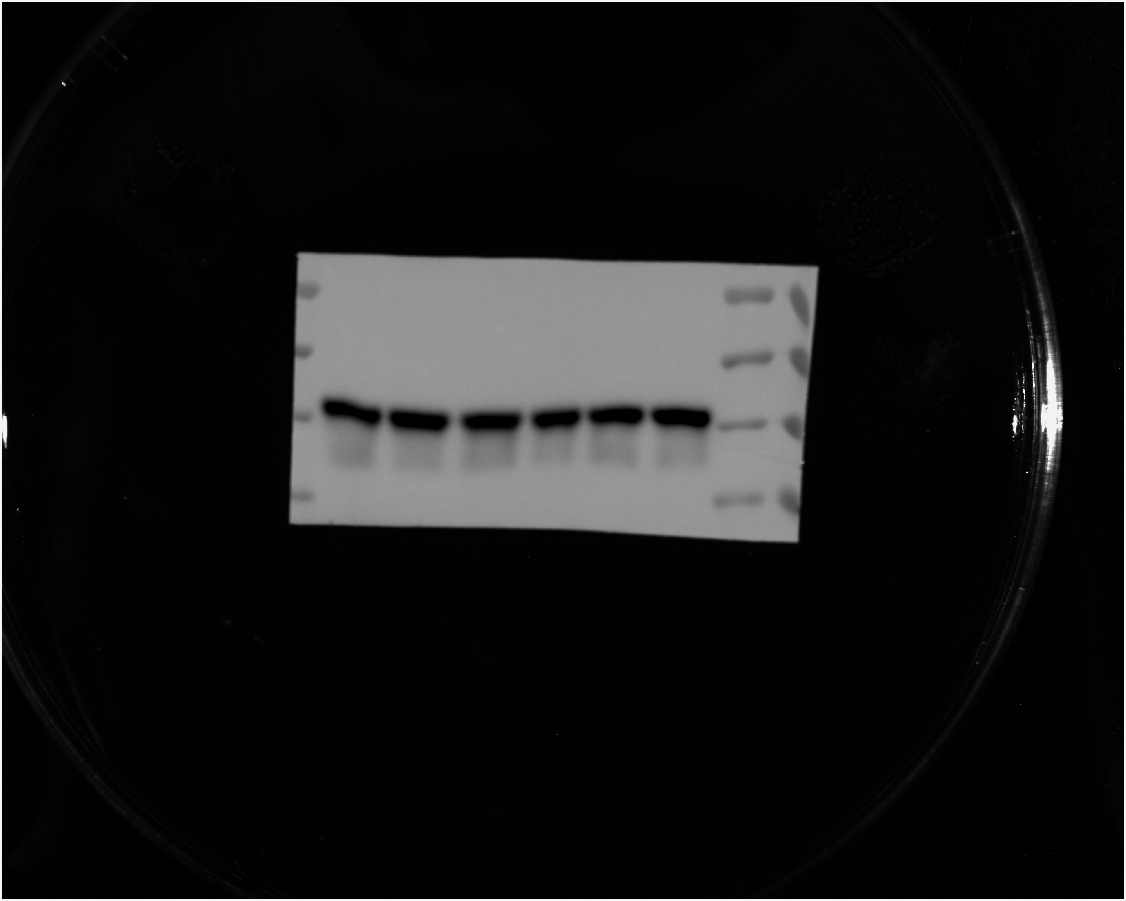

Supplement: Figure 3—figure supplement 4—source data 2. [file elife-103016-fig3-figsupp4-data2.zip › Figure 3-figure supplement 4_Source data 2/β-actin.tif]
